# Synchronous Malignancies Identified by PET-CT Scan in Breast Cancer Patients

**DOI:** 10.5041/RMMJ.10472

**Published:** 2022-04-26

**Authors:** Maya Paran, Katerina Shulman, Boris Kessel, Jasmin Dagan

**Affiliations:** 1 Division of General Surgery, Hillel Yaffe Medical Center, Hadera, Israel; 2 The Ruth & Bruce Rappaport Faculty of Medicine, Technion–Israel Institute of Technology, Haifa, Israel; 3 Oncology Service, Clalit Health Services, Lady Davis Carmel Hospital, Haifa, Israel

**Keywords:** Breast cancer, cancer epidemiology, PET-CT, radiology, screening

## Abstract

Breast cancer is a common malignancy and a common cause of cancer-related mortality in women. Pre-treatment workup of breast cancer does not routinely include positron emission tomography scans. We aimed to review cases of women with breast cancer and a synchronous second primary malignancy. We present three cases of women with non-metastatic cancer in whom a synchronous second primary malignancy was found. Synchronous, second primary malignancies which were identified included rectal cancer, gastrointestinal stromal tumor, and non-small cell lung cancer. All second primary malignancies were identified by a PET-CT scan. In conclusion, PET-CT may be used for detection of secondary primary malignancies in select breast cancer patients.

## INTRODUCTION

Breast cancer is the most commonly diagnosed malignancy and the second most common cause of cancer-related mortality among women in the United States.^1^ Current guidelines recommend screening for breast cancer with early mammography. Additional breast ultrasound and/or magnetic resonance imaging (MRI) are recommended depending on the patient’s age and existing risk factors.^2^,^3^ Screening programs allow early diagnosis and, therefore, have been shown to improve patient outcomes and reduce mortality.^4^

Treatment of early breast cancer is complex and involves a combination of surgery, radiotherapy, and, in selected cases, systemic treatments (chemotherapy, biological and hormonal therapy). The choice of treatment strategy should be based on the tumor burden/location and biology, as well as the age, menopausal status, general health status, and preferences of the patient.^5^

Breast cancer preoperative workup includes basic blood tests, breast and axillary ultrasonography, bilateral mammogram, and biopsy. Consideration of additional imaging for systemic staging including chest and abdominal computed tomography (CT), bone scan, and PET-CT is recommended only in the presence of signs and/or clinical symptoms of metastatic disease.^5^ The current guidelines recommend using PET-CT only in stage IV or recurrent disease. In addition, the use of PET-CT is considered optional in current guidelines in patients with lymph node involvement or HER2-positive disease.^5^ Therefore, no clear recommendation for PET-CT in patients with early breast cancer exists. Moreover, the current guidelines do not recommend considering the use of PET-CT in search of second primary malignancies in breast cancer patients.

The rapidly growing availability of fluorodeoxyglucose (FDG) positron emission tomography (PET)-CT scan presents a relatively new diagnostic possibility and has thus led to changes and modifications of indications for its use over the years. The PET-CT scan is a useful test in different oncology indications, including detection of occult primary malignancy in patients with metastatic disease, cancer staging, assessment of treatment response, and detection of disease recurrence and/or progression.^6^ However, the role of PET-CT in early breast cancer remains unclear, and evidence regarding the advantages of its routine use for initial staging of breast cancer is limited.^7^

Several studies have reported PET-CT to be of no practical value in patients with early breast cancer, (e.g. tumors smaller than 2–3 cm in patients with no palpable nodes).^8^–^11^ Therefore, currently, routine use of PET-CT is not supported by accepted guidelines.^5^

Nevertheless, PET-CT has been suggested to be of value in the detection of second primary malignancies in patients with breast cancer.^12^ Second primary malignancies are classified as either synchronous or metachronous. Synchronous second primary malignancies are tumors that occur within 6 months of the diagnosis of the first malignancy, whereas metachronous malignancies are those which develop 6 months or more after the diagnosis of the primary malignancy.^12^,^13^

Several studies have shown that women with breast cancer have a higher risk of developing a second primary malignancy as compared to the general population.^14^,^15^ Different factors may contribute to the development of secondary primary malignancies, such as previous chemotherapy or radiotherapy, young patient’s age, and hormonal manipulations during adjuvant treatment.^16^,^17^ Most studies have examined the association between treatment methods such as radiotherapy and chemotherapy, and development of second primary malignancy.^16^,^17^ The survival for breast cancer patients with second primary malignancy is significantly poorer.^16^

In this report, we present a case series of patients with non-metastatic breast cancer who underwent a PET-CT scan which revealed a synchronous, aggressive, second primary malignancy.

### Case 1

**Figure 1:**
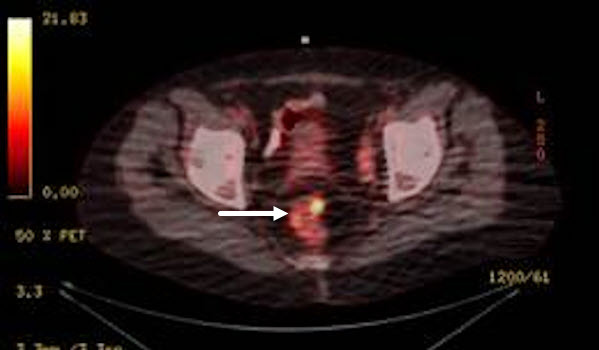
A PET-CT Scan Showing a Rectal Mass.

A previously healthy, asymptomatic 58-year-old woman with no family history of malignancy was evaluated with routine screening mammography. The mammography revealed a 3 cm field of micro-calcifications in her right breast. On physical exam, no palpable mass or enlarged axillary lymph nodes were noted. Breast ultrasonography also revealed no findings. The patient underwent a core biopsy, which was consistent with ductal carcinoma *in situ* (DCIS). The patient completed the accepted preoperative assessment, and, upon her request, PET-CT was performed. The PET-CT scan revealed a 5 cm mid-rectal mass, which was later biopsied (Figure 1). A diagnosis of rectal adenocarcinoma was made, and a full workup was performed, after which the rectal tumor was defined as locally advanced (T4N0M0). The patient received a course of preoperative chemo-radiotherapy and anterior resection, followed by a right lumpectomy.

### Case 2

A 67-year-old woman with a medical history of impaired fasting glucose and dyslipidemia was under follow-up due to a history of breast cancer. The patient had a history of breast cancer in her left breast which was treated with a left lumpectomy followed by chemotherapy and radiotherapy. Eleven years after the initial diagnosis, the patient had a recurrence and was treated with mastectomy and adjuvant chemotherapy. Genetic screening for BRCA was negative. During follow-up, 10 years after the mastectomy, routine mammography demonstrated a 1.4 cm mass in her right breast. Ultrasonography revealed a 1.3 cm mass on the external upper quadrant of the right breast, with no pathologic axillary lymph nodes. A biopsy confirmed the diagnosis of infiltrating ductal carcinoma, ER +3, PR +3, HER2 +2 (FISH negative), Ki-67 40%. The patient had no clinical signs of metastatic disease. The patient completed a PET-CT scan which revealed a heterogeneous large pelvic mass, 9×12 cm. The mass was biopsied, and the results were consistent with a gastrointestinal stromal tumor (GIST). Following a multidisciplinary team discussion, the patient underwent a simultaneous operation on both breast and GIST, which required a segmental small bowel resection. After completion of the abdominal procedure, a right breast lumpectomy with sentinel lymph node biopsy was performed.

### Case 3

A 59-year-old woman with a history of Meniere’s disease and no family history of malignancy was evaluated with routine screening mammography that revealed a suspicious 12 mm mass on her left breast. On breast sonography, a 12 mm irregular mass was identified with no pathologic axillary lymph nodes. On biopsy, the mass was found to be invasive ductal carcinoma grade 2–3, ER +3, PR +3, HER2 negative, Ki-67 index 10%. The patient was further evaluated with a breast MRI and a PET-CT scan. The MRI demonstrated a 19 mm mass on the left breast without axillary lymphadenopathy, and PET-CT revealed a sub-pleural 12 mm mass in the right upper lobe of the right lung. Biopsy from the pulmonary mass was consistent with squamous cell carcinoma of the lung with clinical staging of T1N0M0. After a multidisciplinary discussion, it was decided to operate on the breast first. The patient underwent a lumpectomy and sentinel lymph node biopsy. Following the breast procedure, the patient underwent a thoracoscopic lobectomy of the right lung.

## DISCUSSION

This report presents a case series of women with breast cancer who underwent a PET-CT scan which revealed a synchronous tumor other than breast cancer, including rectal adenocarcinoma, small bowel gastrointestinal stromal tumor (GIST), and non-small cell lung cancer (NSCLC). In all cases, the results of the PET-CT scan, which revealed an aggressive malignancy, led to important and necessary changes in the treatment plan.

Second primary malignancies in women with breast cancer have been previously described in several studies. Research has shown that women with breast cancer have a higher risk of developing a second primary malignancy as compared to the general population.^14^–^18^ For example, as in Case 3 in our series, an association between breast cancer and lung cancer has been previously reported.^19^–^21^ Similarly, an association between breast and colorectal cancer has also been previously reported,^22^ as in Case 1 in this case series. Nevertheless, most studies have reported the incidence of metachronous second primary malignancies after breast cancer,^14^,^16^–^19^ and only a few studies have investigated the rate of synchronous tumors. Metachronous and synchronous malignancies, that might necessitate a change in the treatment plan, may be detected by PET-CT.

The yield of PET-CT in patients with breast cancer for staging has been investigated comprehensively.^7^,^23^–^26^ However, only a small number of studies have investigated the use of this imaging modality for the detection of second primary malignancies in this population. When reviewing the literature, one case report of a woman with breast cancer and an accidentally found second primary malignancy was found.^11^ Of note, the patient in this specific case underwent PET-CT due to a palpable axillary mass.^27^ A retrospective study that investigated the yield of PET-CT in patients with different known primary malignancies, including breast cancer patients, for the detection of second primary malignancies has reported diagnosis of second primary malignancy in at least 1.2% of patients with cancer. The authors reported that the detection of second primary malignancy led to a change in the treatment plan in the majority of cases.^28^ Another study that assessed the detection rate of second primary malignancies in patients undergoing PET-CT as part of a follow-up plan for another primary malignancy reported that a second primary unexpected malignancy was detected in 1.7% of patients.^29^ Moreover, a study that evaluated the benefits of PET-CT for the diagnosis of recurrent breast cancer has reported detecting an incidental second primary malignancy in 4% of patients.^30^ Similarly, a recent study reported that PET-CT revealed a suspected metachronous second primary malignancy in 37 of 233 breast cancer female patients.^12^ When reviewing the cases presented in our report, two of the three cases of second primary malignancies would have been discovered, had the patients undergone the proper screening tests for other malignancies, regardless of the diagnosis of breast cancer. In case 1, the patient was 58 years old at the time of the diagnosis, and thus a routine screening colonoscopy was indicated.^31^ Similarly, in case 3, the patient was 59 years old with >20 pack-year history of smoking and therefore should have undergone a screening low-dose chest CT scan as recommended.^32^

## CONCLUSIONS

The higher incidence of malignancies in patients with breast cancer suggests considering screening these patients in search of synchronous malignancies in select cases, especially in patients who did not complete all relevant screening tests for other malignancies. A possible screening option may be a PET-CT scan, as was used in this report. However, the use of PET-CT as a screening tool must be weighed against the risk of false-positive results which would be followed by unnecessary tests. We believe our results, together with the results of previous studies, suggest a need for future studies to prospectively investigate the role of PET-CT as a screening tool for second primary malignancies in specific patients who are at risk for second primary malignancies. Future, large prospective studies are needed in order to investigate the possible role of PET-CT in the assessment of patients with breast cancer.

## Abbreviations

CT: computed tomography
DCIS: ductal carcinoma *in situ*
FDG: fluorodeoxyglucose
GIST: gastrointestinal stromal tumor
MRI: magnetic resonance imaging
NSCLC: non-small cell lung cancer
PET: positron emission tomography.
